# Association between T2DM and the lowering of testosterone levels among Kashmiri males

**DOI:** 10.20945/2359-3997000000288

**Published:** 2020-08-24

**Authors:** Rabia Farooq, Mohammad Hayat Bhat, Sabhiya Majid, Mohammad Muzaffar Mir

**Affiliations:** 1 University of Bisha College of Medicine Department of Basic Medical Sciences Saudi Arabia Department of Basic Medical Sciences, College of Medicine, University of Bisha, Saudi Arabia; 2 Government Medical College Srinagar Department of Medicine Jammu and Kashmir India Department of Medicine, Government Medical College Srinagar, Jammu and Kashmir, India; 3 Government Medical College Srinagar Department of Biochemistry Jammu and Kashmir India Department of Biochemistry, Government Medical College Srinagar, Jammu and Kashmir, India

**Keywords:** Testosterone, glycosylated haemoglobin, Kashmiri males

## Abstract

**Objective:**

The objective of this study is to study association between testosterone and diabetes in Kashmiri males.

**Subjects and methods:**

A total of 300 males with Type 2 diabetes visited an outpatient and inpatient clinic at Shri Maharaja Hari Singh (SMHS) hospital, Srinagar, J&K India. The blood sugar and HbA1c, which are the markers of diabetes, and sérum testosterone levels were measured. The blood samples from both the cases and controls were collected.

**Results:**

Out of 300 subjects, 42% had a testosterone deficiency. A relationship between type 2 diabetic males and healthy males was observed, and testosterone levels were determined to be significantly lower among diabetic males (p < 0.001) when compared to healthy males. Then, we compared diabetic markers among testosterone deficient and normal testosterone level groups; the mean fasting plasma glucose (p = 0.0019) and glycated haemoglobin (HbA1c; p = 0.0449) levels were significantly higher in the testosterone deficient group than in the control group. To elucidate the relationship between the serum total testosterone level and fasting plasma glucose and HbA1c values, Pearson's correlation test was performed. Fasting plasma glucose levels (r = −0.252, p = 0.001) and HbA1c values (r = −0.697, p = 0.001) showed a significant negative correlation with serum testosterone levels among diabetic males.

**Conclusions:**

This study shows that diabetes causes low testosterone levels among males, and lower testosterone levels can act as a marker for diabetes. Thus, with timely intervention, mortality and co-morbidity associated with diabetes can be prevented.

## INTRODUCTION

Diabetes mellitus is considered to be the epidemic of the 21st century, which affected more than 422 million people in 2014. According to the International Diabetes Federation (IDF), 463 million people are diabetic worldwide, and 1 in 2 adults remains undiagnosed (1). Among various types of diabetes, Type-2 diabetes mellitus (T2DM) is most prevalent; it affects approximately 5.9% of the world's population. In India, which is the diabetic capital of the world, it has been reported that approximately 62.4 and 77.2 million people are diabetic and prediabetic, respectively (2). Various studies have revealed a connection between male hypogonadism and diabetes (3,4). Hypogonadism is defined “as a clinical syndrome associated with biochemical evidence of testosterone deficiency”, and it usually remains undiagnosed (5,6). A Massachusetts male aging study has reported that 75 out of 100 males with diabetes have low testosterone levels. These males are at a risk of developing ED (7). Numerous studies have observed lower testosterone levels in men (hypogonadism) with T2DM (8). In 2011, a study on 1292 middle-aged and old men in England have determined that both sex hormone binding globulin levels and testosterone were linked with hyperglycaemia (1). Similarly, numerous studies have observed lower levels of testosterone in diabetic men compared with healthy controls (2).

**Figure 1:**
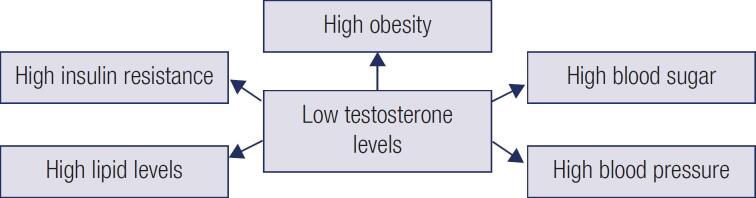
Effect of low testosterone levels on various systems in the body.

Insulin resistance is the main risk factor for developing this type of diabetes, which is also caused by central obesity and previously upper abdominal adiposity (9,10). Many studies have shown an inverse relation between free testosterone and the degree of obesity, which means that low levels of testosterone are observed among obese males (11,12). It is known that obesity causes the conversion of testosterone to estradiol by enzyme aromatase, which further lowers testosterone levels (13). A previous study on diabetic males has reported that an antidiabetic drug rosiglitazone increases testosterone levels (14). In fact, testosterone replacement in people with diabetes and hypogonadism improves insulin sensitivity and glucose homeostasis (15-17). However, some studies have reported that testosterone administration does not affect blood sugar control among hypogonadic diabetic patients (18,19). Glycosylated haemoglobin (HbA1C), which is a measure of hyperglycaemia, IR, and HbA1C are correlated with atherosclerosis and coronary heart disease in men. However, it is not known whether low testosterone levels are the source or consequence of developing diabetes or metabolic syndrome (Figure 1).

Therefore, we studied the prevalence of testosterone deficiency in subjects with type 2 diabetes and assessed the relationship between testosterone deficiency and diabetic markers to understand the role of diabetes on lowering testosterone levels among males. Thus, by accessing testosterone levels prior to the development of diabetes, it may be possible to reduce the risk of developing this disease and provide timely intervention to decrease comorbidity.

## SUBJECTS AND METHODS

We performed the study at the Department of Biochemistry, Government Medical College Srinagar. The participants were 20-60-year-old men who came in for a hormone profile investigation or a diabetic check-up at the outpatient department (OPD)/inpatient department (IPD) at the Shri Maharaja Hari Singh hospital (SMHS), Srinagar. The cases were confirmed by senior endocrinologist. Approximately 300 samples were collected over the period of 2 years. A proper informed consent both in English and vernacular languages was obtained from the participants. All ethical guidelines were taken into consideration. The study was approved by the Departmental Ethical Committee of Government Medical College Srinagar vide No: Bio/GMC/2241A/2017 dated: 11-11-2017.

Inclusion criteria: 1. Diabetic 20-60-year-old males; 2. Males from the Kashmir valley.

Exclusion criteria: 1. Non-diabetic males; 2. Males older than 60 years of age; 3. Males of non-Kashmiri origin.

A total of 310 healthy males were used as controls. A proper questionnaire was formulated, which included anthropometric parameters [e.g., weight, height, body mass index (BMI), waist–hip ratio (WHR)], biochemical parameters [e.g., fasting blood sugar (FBS), post prandial (PP), lipid profile, insulin levels, HbA1C), and hormone profile, which mainly includes testosterone levels. Other factors (e.g., baldness, decrease in sexual desire, erectile dysfunction, and physical activity duration) were taken into consideration. Height, weight, and waist circumference were measured in standing subjects wearing light clothing without shoes; waist was measured at the umbilical line according to a written protocol. BMI (kg/m^2^) was calculated. The waist-hip ratio (WHR) was used as an integrated measure of obesity and fat distribution.

Patients who came in for a normal routine check-up and whose HbA1c and testosterone levels were normal were used as controls. Males suffering from any other disease, which affects their hormone profile, were excluded from the study.

In the morning, fasting blood samples were taken from both the cases and controls in both heparinized and non-heparinized vials. Hormone profile was obtained by an automated electrochemiluminescence immunoassay analyser (ECLIA) (Abbot); a semi-automated analyser (Siemens) was used for normal biochemical investigations.

The obtained data were analysed using the Graph pad prism. The values were expressed as the mean ± standard deviation (SD) and assessed using Student's t-test (independent and unpaired). Pearson's correlation coefficient was used to determine the correlation between serum testosterone with FPS and HbA1c. All statistical tests were two-tailed with statistical significance defined as p < 0.001.

## RESULTS

The anthropometric characteristics of both the cases and controls are shown in Table 1. Significant differences were observed between BMI, WHR, systolic blood pressure (SBP), and diastolic blood pressure (DBP) when cases were compared with the control group. The mean age of participants was 52 years old; the mean BMI, SBP, and DBP were 26 kg/m^2^, 146 mmHg, and 88 mmHg, respectively.

**Table 1:** Anthropometric characteristics of diabetic cases and controls

Parameters	Cases (n = 300)	Controls (n = 310)	P value
Age (years)	52 ± 11	50 ± 15	--
BMI (kg/m^2^)	26 ± 3	20 ± 4	<0.0001
SBP (mmHg)	146 ± 17	116 ± 16	<0.0001
DBP (mmHg)	88 ± 14	77 ± 9	<0.0001
WHR (cm^2^)	1.1 ± 0.4	0.8 ± 0.1	<0.0001

Table 2 shows the baseline characteristics between the cases (diabetic) and healthy individuals; highly significant (p) differences were observed (p < 0.0001). The mean levels of fasting plasma glucose, HbA1c, and serum insulin were 139.0 mg/dL, 8%, and 9 μIU/mL, respectively. The mean level of total testosterone was determined to be 3.9 ng/mL, which was lower than that in the normal control group and was statistically significant (p < 0.0001).

**Table 2:** Baseline characteristics of the cases and controls

Parameters	Cases (n = 300)	Controls (n = 310)	P value
FBS (mg/dL)	139 ± 21.2	95 ± 18	<0.0001
PP (mg/dL)	264 ± 46	139 ± 9	<0.0001
Insulin (IU/mL)	9 ± 1.0	12.2 ± 1.5	<0.0001
HbA1C %	8 ± 2.5	5.2 ± 1.5	<0.0001
Testosterone (ng/mL)	3.9 ± 1.2	6 ± 2.5	<0.0001

Depending upon serum testosterone levels, diabetic males were further categorized into testosterone deficient and normal testosterone level groups. The percentage of testosterone deficient diabetic males was determined to be 42%. The parameters related to glycaemic control (i.e., fasting plasma glucose and HbA1c) were determined to be significantly higher in the testosterone deficient group compared with the normal testosterone diabetic group (p < 0.05). In the testosterone deficient group, the mean SBP and DBP were 138 mmHg and 82 mmHg, respectively (Table 3). The mean levels of fasting plasma glucose and HbA1c were 145 mg/dL, 9.2%, and 9 μIU/mL, respectively (Table 3).

**Table 3:** Baseline characteristics among testosterone deficient and normal groups

Parameters	Testosterone deficient (N = 126)	Testosterone normal (N = 174)	P value
FPG (mg/dL)	145 ± 35	135 ± 20	0.0019
HbA1C%	9.2 ± 2.5	8.7 ± 1.8	0.0449
SBP (mmHg)	138 ± 12.2	133 ± 10	<0.0001
DBP (mmHg)	82 ± 13	79 ± 10	0.0246

**Figure 2:**
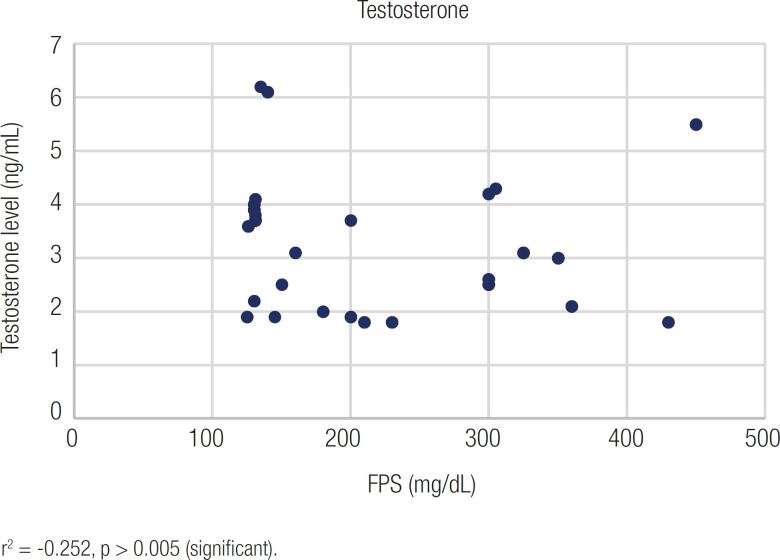
Representative figure showing the correlation between FPG and serum total testosterone among diabetic patients.

**Figure 3:**
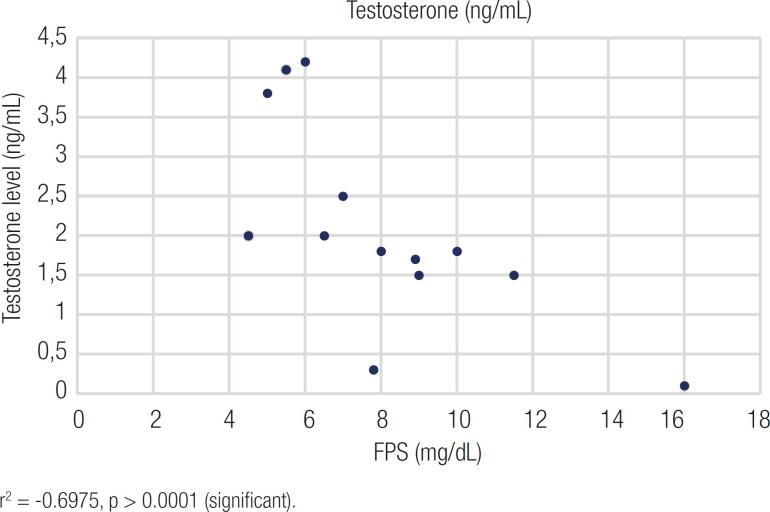
Representative figure showing the correlation between HbA1c% and serum total testosterone among diabetic patients.

Figures 2 and 3 show the scatter diagrams of the serum total testosterone level, fasting plasma glucose (FPG), and glycated haemoglobin (HbA1c). Subsequently, Pearson's correlation coefficients were calculated to evaluate the relationship between fasting plasma glucose, HbA1c, and serum total testosterone values. Figure 2 shows that Pearson's correlation coefficient (r) between total testosterone and fasting plasma glucose levels was −0.252, and the P value was >0.005 (significant). Pearson's correlation coefficient (r) between total testosterone and HbA1c levels was −0.697, and the P value was >0.0001 (Figure 3).

## DISCUSSION

In many studies, low testosterone levels are determined to be associated with the high prevalence of MetS (20-22). MetS includes a cluster of diseases such as Type 2 diabetes mellitus. A change in the serum testosterone level among males causes insulin resistance, obesity, high blood pressure, and the presence of MetS; reverse may be also possible. In a study, which included 651 Finnish males, MetS and lower testosterone levels were observed (23). In a study among Korean population, the prevalence of testosterone deficiency was determined to be 34.9% among diabetic males (24).

In this study, we observed that the serum testosterone levels were negatively correlated with fasting plasma glucose levels and HbA1c values, which was consistent with other studies.

In previous studies, serum testosterone levels were negatively correlated with fasting plasma glucose levels, HbA1c values, and insulin sensitivity (23,25,26). Insulin resistance among males was determined to be associated with lower testosterone levels. Previous studies have demonstrated that insulin resistance was associated with low serum testosterone levels in men because testosterone controls the glycogen synthesis system in muscle (27-29). Mortality is another outcome, which is affected by the serum testosterone level. Low serum testosterone levels, including both serum total and free testosterone, were determined to be associated with increased mortality among 858 old males (30). It can be suggested that glycaemic control may modulate testosterone levels by either affecting glycogen synthesis or hypogonadism among diabetic men; this process is related to kisspeptin (a neuropeptide), which has been observed to be down-regulated with hyperglycaemia in various animal studies (31). In fact, kisspeptin administration among hypogonadic males increased the secretion of luteinizing hormone (LH), which also increased endogenous testosterone secretion. These studies relate hypogonadotropic hypogonadism and diabetes (32-35). Heufelder and cols. (36) have proposed the beneficial effects of testosterone administration on insulin resistance in patients with diabetes.

However, some studies have shown contradictory results, i.e., positive correlation between total testosterone levels and HbA1c levels but no correlation with fasting plasma glucose (37).

### Limitation

The study was a single hospital-based study, and the number of participants is not representative of Kashmiri male population. In addition, in this study, free testosterone and bioavailable testosterone levels are not accessed. Thus, further studies are required with a larger sample size to establish the role of testosterone on the glycaemic status of male patients with type 2 diabetes mellitus.

In conclusion, this study suggests that lowered testosterone levels can considerably increase the risk of T2DM among males. Variations in the serum testosterone levels can be used as a biomarker to assess the progression of T2DM. In addition, testosterone supplementation should be considered to improve clinical outcomes among diabetic men.
